# Electrochemical properties of Sn-decorated SnO nanobranches as an anode of Li-ion battery

**DOI:** 10.1186/s40580-016-0070-1

**Published:** 2016-05-01

**Authors:** Jeong Ho Shin, Jae Yong Song

**Affiliations:** Korea Research Institute of Standards and Science, Materials Genome Center, Daejeon, 34113 South Korea

**Keywords:** SnO, Nanobranch, Electrochemical property, Phase transformation

## Abstract

Sn-based oxide materials as an anode of lithium ion batteries (LIBs) suffer from the unavoidable mechanical stress originated from huge volume changes during lithiation/delithiation reactions. We synthesized the hierarchical SnO nanobranches (NBs) decorated with Sn nanoparticles on Cu current collector using a vapor transport method. The Sn-decorated SnO NBs as an anode of LIB showed good electrochemical performance with high reversible capacity retention of as high as 502 mAh/g and rate capability of 455 mAh/g at a current density of 2.0 A/g after 50 cycles. Through the morphological and crystal structure analyses after the charge and discharge processes, it was found that the morphology of Sn-decorated SnO NBs was transformed to nanoporous layered-structure, composed of Sn and lithium oxide, during the repeated lithiation/delithiation reactions. The free-volume of Sn-decorated SnO NBs and nanoporous layered-structure effectively accommodate the huge volume changes and enhance the electrochemical cyclability by facilitating the diffusion of Li-ions.

**Electronic supplementary material:**

The online version of this article (doi:10.1186/s40580-016-0070-1) contains supplementary material, which is available to authorized users.

## Background

Rechargeable lithium ion batteries have been widely used as energy sources for portable electronic devices such as cellular phones, cameras, and lap-top computers. LIBs are also considered one of the most promising candidates for a next generation large-scale power source, i.e., electric vehicles (EVs) or hybrid electric vehicles (HEVs), because of their high energy density and good cyclability [1]. However, many critical challenges related to the improvement of LIB performance must still be addressed [2, 3]. Among them, the energy density of the anode should be improved to meet the increasing demand for LIBs with higher energy density and power density. Although graphite has been commercialized as an anode material of LIBs due to its low operating voltage and excellent cyclability, its Li storage capacity is limited to the theoretical maximum capacity of 372 mAh/g [4]. Consequently, extensive studies have focused on developing new anode materials to replace graphite [5–7]. Among various anode materials, Sn has been investigated as an alternative candidate due to its high theoretical capacity of 994 mAh/g [8]. Unfortunately, it shows very poor cyclability due to deterioration of the anode caused by the huge volume expansion of 360 % during the Li-Sn alloying process [9]. In order to overcome the anode failure related to the volume expansion, the use of a free-volume accommodating the volume expansion of the anode materials was suggested, e.g., in the form of nanowires, nanotubes, nanoparticles, porous structures, etc. [10–15]. Another approach is to prepare a composite structure of an active component in the inactive matrix, where the inactive matrix plays a role of accommodating the volume expansion of the active component during the lithiation process [16]. As the lithiation of Sn-based oxides such as SnO and SnO_2_ results in a composite structure of inactive lithium oxide and active Sn phases, the lithium oxide matrix helps to mitigate the large volume expansion. Generally, it is easier to synthesize the SnO_2_ phase than the SnO phase, because the latter is a metastable and low-temperature phase in the binary Sn–O system [17]. Nevertheless, many researchers have investigated various SnO nanostructures such as nanoparticles, nanosheets, nanoflowers, etc. as anode materials, because the theoretical capacity (875 mAh/g) of SnO is much higher than that (783 mAh/g) of SnO_2_ [18–21]. However, these fabrication methods require a binder and conductive carbon source, which reduces the anode capacity. Therefore, we were motivated to directly synthesize Sn nanoparticles (NPs) decorated SnO nanobranches (NBs) on a Cu current collector at 503 K without any additives. Herein, the electrochemical characteristics of the SnO NBs as an anode of LIBs were investigated together with the phase transformation and structural analysis.

## Methods

The Sn-decorated SnO NBs were prepared by a vapor transport method. Cu foil (18 μm in thickness, 99.8 at %, Nippon Foil Mfg Co.) was placed at the center of horizontal quartz tube which was evacuated to 5 × 10^−3^ torr. The temperature of the tube center was set to 503 K. Sn vapors evaporated from a resistance-heated tungsten boat were supplied by Ar carrier gas (99.9 %). The growth time of SnO NBs was 2 h. The detailed process can be found in the previous report [20]. The SnO film was synthesized by the same vapor transport method. When the mixture gas of Ar and O_2_ (Ar:O_2_ = 98:2) was used instead of the Ar gas, SnO thin film (500 nm in thickness) grew on Cu substrate for 30 min. The electrochemical properties of SnO NBs and SnO film as an anode of LIBs were investigated using a CR2032 coin cell. As a counter electrode, Li metal foil was used. The separator was microporous polyethylene (Celgard 2400, Celgard LLC. Charlotte, NC, USA) and the electrolyte was 1 mol/L lithium hexafluorophosphate in a 1:1 (v/v ratio) mixture of ethylene carbonate and diethyl carbonate (Techno Semichem). The fabrication was conducted in Ar-filled glove box. The weights of all samples were measured using a microbalance (METTER TOLEDO, AT261). Cyclic voltammogram (CV) curves were measured in the potential range of 0.001 to 2.5 V with a scan rate of 0.1 mV/s at room temperature using an electrochemical analyzer (Solartron 1280z). Galvanostatic charge/discharge measurements were carried out in the potential range of 0.001 to 2.5 V at the constant current of 0.1 A/g using a multi-channel potentiostat/galvanostat (VMP3, Biologic). The C-rate was measured by varying the currents as 0.1, 0.2, 0.5, 1.0, and 2.0 A/g. The microstructures and crystal structures were analyzed using an X-ray diffraction (XRD. Cu-Kα, Bruker D8), field emission scanning electron microscope (SEM, Hitachi S4800), and field emission transmission microscope (TEM, FEI Tecnai F30).

## Results and discussion

**Fig. 1:**
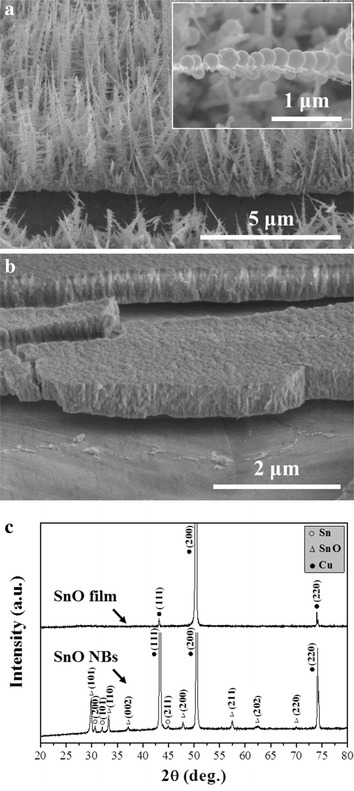
Typical SEM image of (**a**) SnO NBs and (**b**) SnO film in the as-prepared state. The *inset* in **a** shows a high-magnified SEM image. **c** Typical XRD patterns of SnO NBs and SnO film in the as-prepared state

Figure 1 shows SEM morphologies and XRD patterns of as-prepared Sn-decorated SnO NBs and SnO film, which were grown on Cu foil, respectively. The SnO NBs with a length of approximately 5 μm had a hierarchical structure of a backbone and secondary and tertiary branches, as shown in Fig. 1a. In the inset of Fig. 1a, the enlarged SEM image indicates that Sn nanoparticles (NPs) of 100 to 200 nm in diameter were decorated on the surface of the SnO NBs. The spherical nanoparticles were indexed to be a tetragonal crystal structure of metal Sn, as confirmed by TEM analyses (see Additional file 1: Figure S1). The Fig. 1b shows a typical SEM image of as-prepared SnO film with a thickness of 500 nm. The XRD results show that the NBs were composed of SnO (JCPDS#85-0712) and Sn (JCPDS#86-2262), as shown in Fig. 1c. The SnO film was analyzed to be an amorphous phase. A more detailed description of the structural characteristics can be found in our previous report [22].

**Fig. 2:**
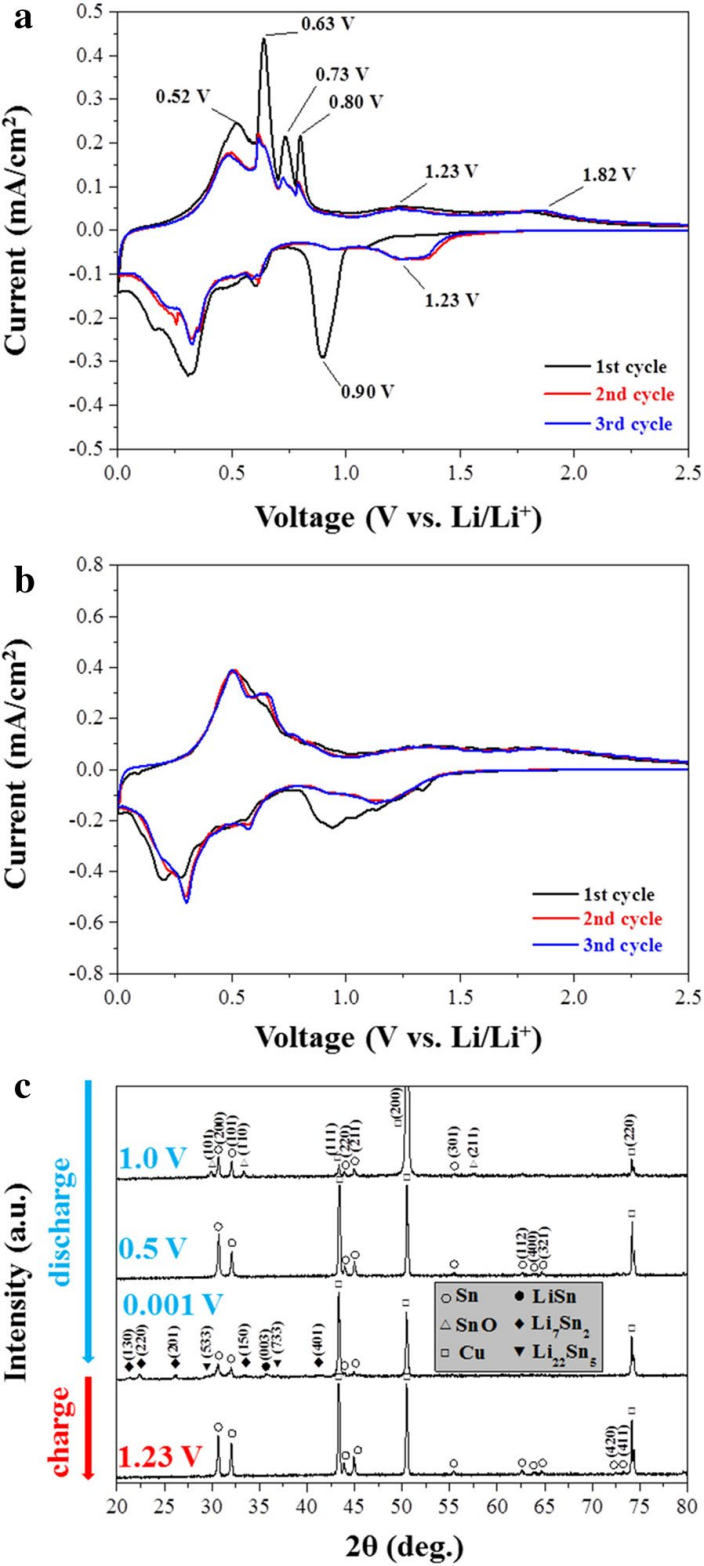
Cyclic voltammetry (CV) profiles of (**a**) SnO NBs and (**b**) SnO film, **c** XRD patterns of the SnO NBs with the reaction potential with Li^+^ in the first charge and discharge cycle

In order to get insight into the reaction behaviors of the SnO NBs with Li^+^ during the charge/discharge processes, cyclic voltammetry (CV) and ex situ XRD measurements were carried out. Figure 2a shows the first three CV curves of the SnO NBs in a voltage range of 0.001 to 2.5 V at a scan rate of 0.1 mV/s. In the discharge process of the first cycle, the sharp cathodic peak at 0.9 V indicates that the SnO NBs reacted with Li^+^ to form Li_2_O and Sn as described in Eq. (1).

1 $${\text{SnO }} + \, 2{\text{Li}}^{ + } + \, 2{\text{e}}^{ - } \to {\text{ Sn }} + {\text{ Li}}_{2} {\text{O}}$$

Since the Gibbs free energy change of Eq. (1) is given as ΔG = −256.8 kJ/mol at 298 K, the reaction can spontaneously occur [23]. Here, the formation of Li_2_O is generally believed to be irreversible. With continuous discharging processes, several cathodic peaks appeared at 0.60, 0.30, and 0.16 V, in sequence. These peaks were due to the lithiation reaction with forming Li_x_Sn. The reactions can be given as follows:

2 $${\text{Sn }} + x{\text{Li}}^{ + } + x{\text{e}}^{ - } \leftrightarrow {\text{ Li}}_{\text{x}} {\text{Sn }}\left( {0 \le x \le 4.4} \right)$$

In the charge process, a series of conspicuous oxidation peaks appeared at the voltages of 0.52, 0.63, 0.73, and 0.80 V, corresponding to the delithiation of Li_22_Sn_5_, Li_7_Sn_2_, Li_7_Sn_3_, and LiSn phases, respectively [24]. With further oxidation of charging process, a broad peak occurred at 1.23 V due to the formation of Sn phase from the LiSn alloy [25]. Therefore, the Li-Sn alloying and dealloying reactions occurred reversibly, as described by Eq. (2). This result coincided with the charge/discharge profiles of SnO NBs in the voltage range of 0.001 V to 2.5 V at the current density of 0.1 A/g (see Additional file 1: Figure S2a). Except for the first discharge process, the subsequent charge/discharge curves showed highly reversible behavior. The irreversible discharge capacity (about 190 mAh/g) of the first cycle was ascribed to the formation of Li_2_O and Sn from SnO phase according to Eq. (1). Figure 2b shows the CV curve of the SnO film in comparison with the electrochemical behaviors of the SnO NBs. Interestingly, it was found that the representative sharp and stepwise reaction peaks (cathodic and oxidation peaks) observed in Fig. 2a did not appear in the CV curves of SnO film. The reaction peaks during the first cycle of SnO film appeared sharper than those during subsequent cycles, although the peak potentials of SnO films were equal to those of SnO NBs. The broad reaction peaks of the subsequent cycles indicated that the film structure without a free-volume suppressed the volume expansion and then the reaction of Eq. (2) was difficult to occur. Therefore, the average capacities of SnO films were 40 % lower than those of SnO NBs during the initial five cycles, as shown in the charge/discharge profiles (see Additional file 1: Figure S2b).

This might be attributed to the morphological difference between nanobranch and film structures. That is, the hierarchical structures of the SnO NBs with free-volume provide the Li ions with short diffusion path and facilitate the sequential Li-Sn alloying and dealloying reactions, while the dense structure of SnO film plays a role of diffusion barrier. On the other hand, a weak and broader oxidation peaks were observed near 1.82 V in Fig. 2a. This was ascribed to the formation of SnO phase [26]. According to the in situ Mössbauer spectroscopy experiment of Dahn and co-workers, SnO could be regenerated to some extent by the reaction of Sn and oxygen, which was subsequently liberated due to the reduction of Li_2_O in the charging process [27]. In the subsequent cycles (second and third cycles), the alloying and dealloying reactions almost coincided with those of the first cycle, despite the decreasing peak intensities and slight shift of peak voltages. It is noted that the peak at 0.90 V disappeared and a new broad peak appeared near 1.23 V in the discharge process. This agreed well with previous reports that the broad cathodic peak near 1.23 V represent the formation of lithium oxide from the SnO phase formed around 1.82 V in the charging process [25, 26, 28]. This agreed with the result that the cathodic peak at 1.23 V and the oxidation peak at 1.82 V reversibly appeared in the second and third cycles.

Figure 2c shows the variations of XRD patterns of the SnO NBs with the discharge and charge voltages of the first cycle. After discharging up to 1.0 V, the overall peak intensities were reduced in comparison with those in the as-prepared state (Fig. 1c). This was related to the decomposition reaction of Eq. (1). When the discharging process was conducted up to 0.5 V, the XRD peaks of SnO phase disappeared and only the peaks of Sn phase remained. This is attributed to the complete reduction of SnO to Sn and Li_2_O phases, which agrees with the increased peak intensities of the Sn phases. The XRD peaks of the Li_2_O phase were not observed due to its amorphous character. With further discharging up to 0.001 V, various XRD peaks of Sn and Li_x_Sn alloys such as LiSn, Li_7_Sn_2_, and Li_22_Sn_5_ phases appeared. Despite the complete discharging process, the appearance of LiSn, Li_7_Sn_2_ and Sn phases indicates that incomplete lithiation of SnO NBs occurred in the first cycle. After charging up to 1.23 V in the first cycle, the XRD peaks of Li-Sn alloys completely disappeared and only the XRD peaks of Sn phase existed following the reversible reaction of Eq. (2).

**Fig. 3:**
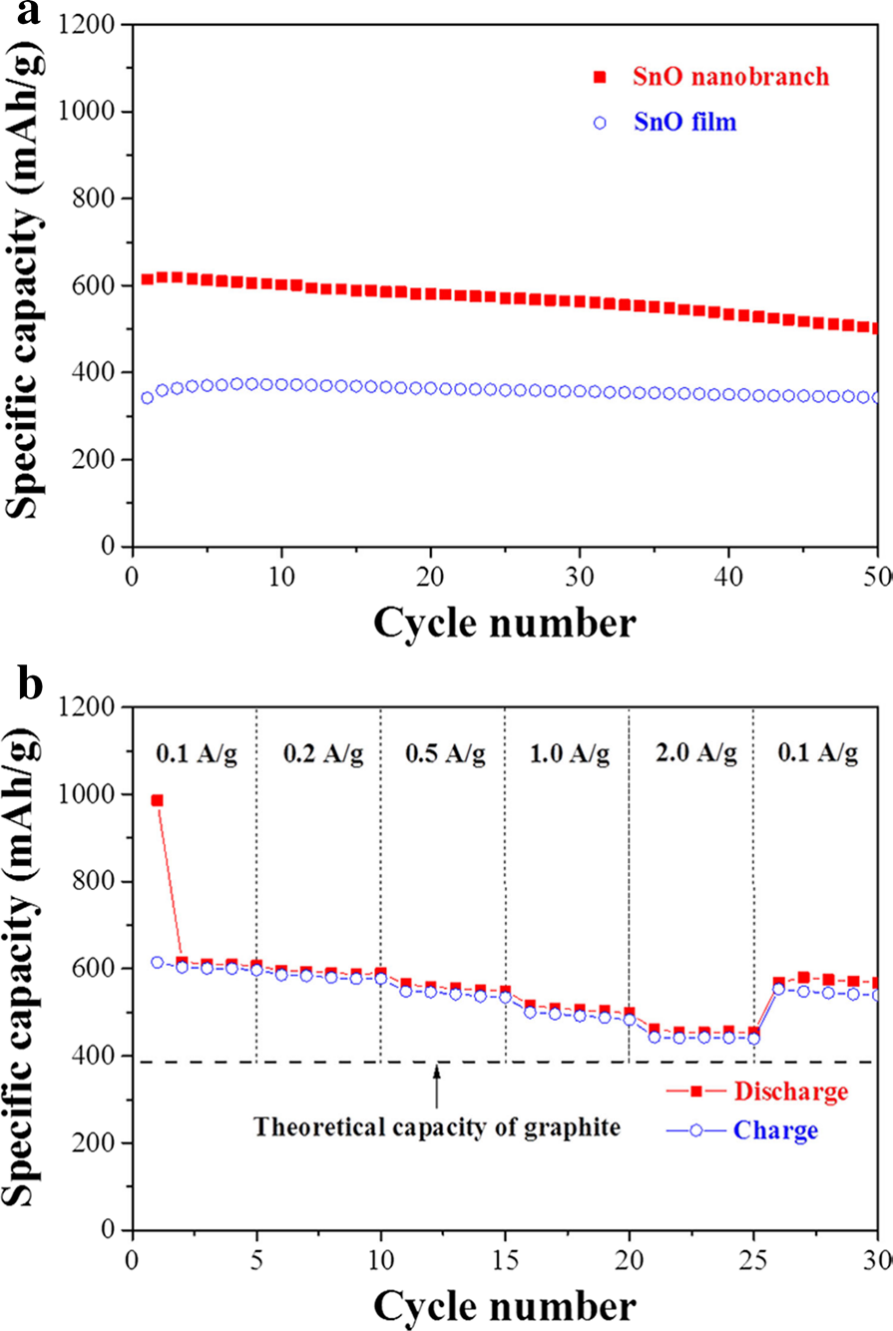
Variations of (**a**) specific capacities with charge/discharge cycle number and (**b**) rate capability of SnO NBs in cut-off voltage range of 0.001 to 1.0 V

Galvanostatic charge/discharge tests were conducted to evaluate the electrochemical performance of Sn-decorated SnO NBs as anode materials for LIBs. Figure 3a shows the cycling properties of SnO NBs and films during 50 cycles. The cyclability was measured between 0.001 and 1.0 V at the current density of 0.1 A/g. The SnO film exhibited capacity retention of 342 mAh/g after 50 cycles with the maximum capacity of 375 mAh/g. It is notable that the SnO NBs showed much higher reversible capacity retention of 502 mAh/g up to 50 cycles with a stable cycling performance. Even though the specific capacity of SnO NBs slowly decreased with the number of cycles, their cyclability and capacity retention values were comparable to those of other nanostructured SnO anode materials [18–21]. Therefore, it is supposed that the higher capacity of the SnO NBs is attributed to: (i) the large amount of Sn nanoparticles existing on the surface of SnO NBs and (ii) the branched nanostructures and nanoporous layered structure with free-volume facilitating faster Li^+^ diffusion and accommodating the volume expansion caused by lithiation.

The rate capability of the SnO NBs was also evaluated with variation of the current densities from 0.1 to 2.0 A/g. The SnO NBs exhibited excellent rate capability, as shown in Fig. 3b. The reversible capacities at voltage of 1.0 V were observed in the range of 616–455 mAh/g with a Coulombic efficiency of 97 %. This was much higher than the theoretical capacity (372 mAh/g) of graphite.

**Fig. 4:**
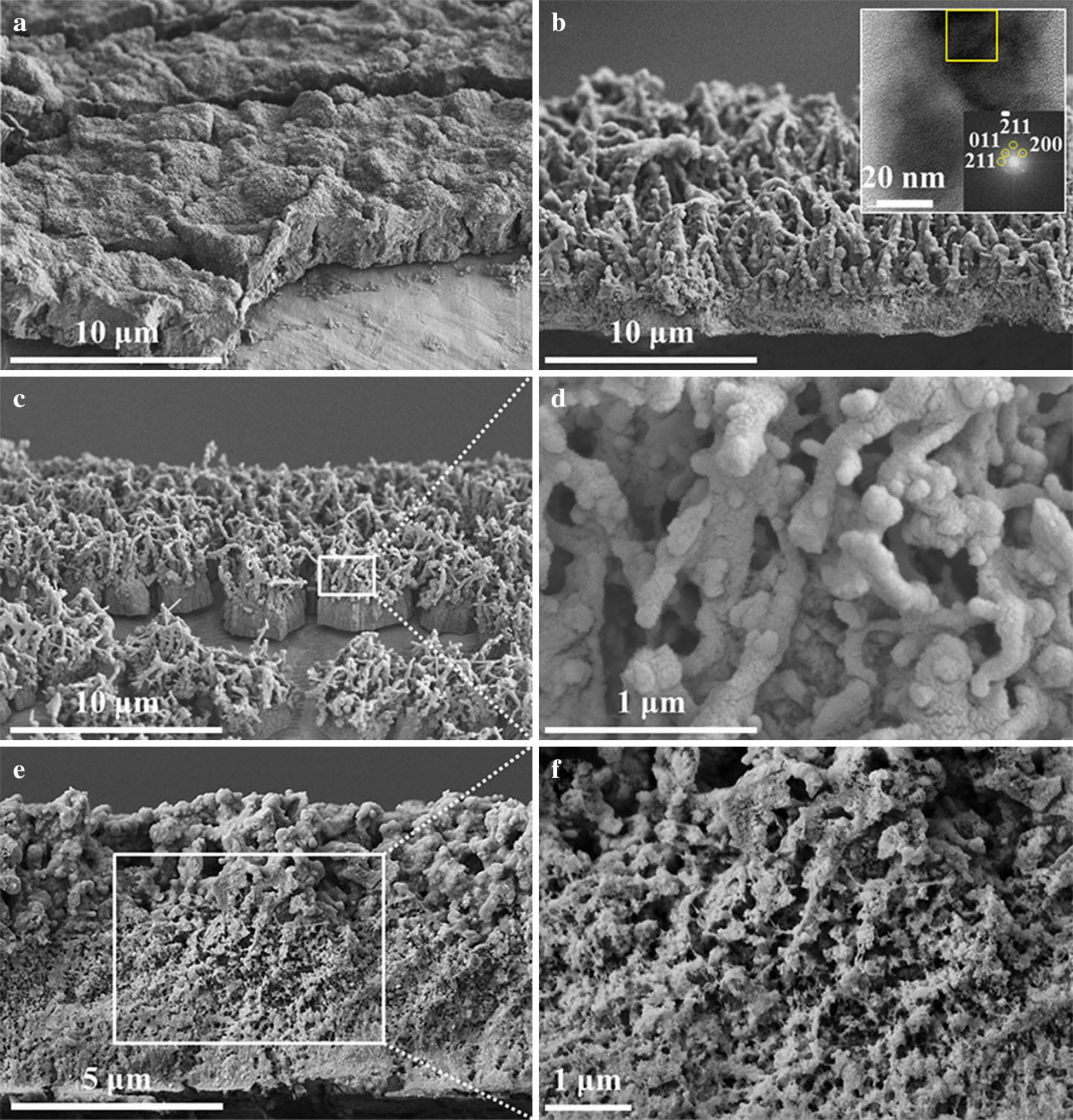
Morphological changes of SnO film and SnO NBs with charge/discharge cycling processes. Cross-sectional SEM image of (**a**) SnO film after 50 cycles and (**b**) SnO NBs after the first cycle. The *insets* in **b** show an HR TEM image and the corresponding FFT image of a hierarchical SnO NB after the first cycle. **c** SEM image of SnO NBs after 10 cycles, **d** magnified SEM image of the area marked in **c**, **e** cross-sectional SEM image of SnO NBs after 50 cycles, (**f**) magnified SEM image of the area marked in (**e**)

Figure 4 shows the typical microstructural changes of SnO film and SnO NBs with charge/discharge cycles at a rate of 0.1 A/g. As shown in Fig. 4a, SnO film still had a dense layered structure after 50 cycles. In comparison, the hierarchical structure of Sn-decorated SnO NBs remained more or less after the first cycle, even though they were thickened due to the lithiation (Fig. 4b). The inset of Fig. 4b show a high-resolution TEM image of a Sn-decorated SnO NB after the first cycle and a corresponding Fast Fourier Transform (FFT) image of the marked area. The FFT image was indexed to the tetragonal crystal structure of metal Sn. This indicates that the SnO NBs were transformed to a composite structure of an amorphous lithium oxide and nanosized Sn although lithium oxide phase was not indexed due to its amorphous character. After 10 cycles, the branched nanostructure still remained at the top side while nanoporous structure appeared at the bottom (Fig. 4c). Figure 4d shows the higher magnified SEM image of the area marked by the white rectangle in Fig. 4c. It was noted that some of the branched nanostructures agglomerated. After 50 cycles, the SnO NBs were fully transformed to highly nanoporous layered structure (Fig. 4e). As shown in the magnified image (Fig. 4f), the porous layer was composed of networked nanopores (several tens of hundreds of nanometers in diameter). The microstructural transformation from hierarchical nanobranches to a nanoporous layered structure might be responsible for the high reversible capacity retention of the SnO NBs, as shown in Fig. 3. Although the original morphology of the SnO NBs was completely transformed to the networked nanoporous layer, the porous structure still has significant specific surface area and free volume.

Therefore, it is supposed that the higher electrochemical performance of the SnO NBs, in comparison with SnO films, can be attributed to: (i) morphological advantages i.e., branched nanostructures and nanoporous layered structure including large free-volume, which provides fast diffusion channels for Li^+^ and accommodates the huge volume changes following the Li-Sn alloying and dealloying reactions [29]; (ii) the inactive lithium oxide that forms at the voltage of 0.9 V during the first discharge, mitigates the volume expansion [30]; and (iii) Sn NPs decorated on the surface of SnO NBs increases the electrochemical capacity [31].

## Conclusions

Sn-decorated SnO NBs were directly synthesized on a Cu foil by a vapor transport process. The SnO NBs as an anode of a LIB exhibited a high reversible capacity of approximately 502 mAh/g up to 50 cycles as well as a high rate capability of 455 mAh/g at a rate of 2.0 A/g. The excellent electrochemical performance of Sn-decorated SnO NBs is attributed to the free-volume of the nanostructures as well as the decorated Sn nanoparticles. Microstructural studies revealed that, during the charge/discharge processes, the nanobranches transformed to nanoporous layered structure. Both nanostructures, which include free-volumes, facilitates fast Li^+^ diffusion and accommodates the huge volume changes. It is suggested that hierarchical and/or nanoporous nanostructures will be one of potential ways in enhancing the electrochemical performance of other anode materials of LIBs.

## Additional file

**Additional file 1.** Electronic supporting information includes additional TEM images and electrochemical properties.
