# Supplementary material for: Electrochemical properties of Sn-decorated SnO nanobranches as an anode of Li-ion battery
Source: Nano Converg. 2016 May 1;3:9. doi: 10.1186/s40580-016-0070-1 (PMC5271146; doi:10.1186/s40580-016-0070-1)
Supplement: Supplementary file 1 — Additional file 1. Electronic supporting information includes additional TEM images and electrochemical properties. [file 40580_2016_70_MOESM1_ESM.docx]

**Electronic Supporting Information**

**Electrochemical Properties of Sn-Decorated SnO Nanobranches as an Anode of Li-Ion Battery**

Jeong Ho Shin and Jae Yong Song*,

Korea Research Institute of Standards and Science, Materials Genome Center, Daejeon, 34113, South Korea

*To whom all correspondences should be addressed: jysong@kriss.re.kr (J.Y. Song)


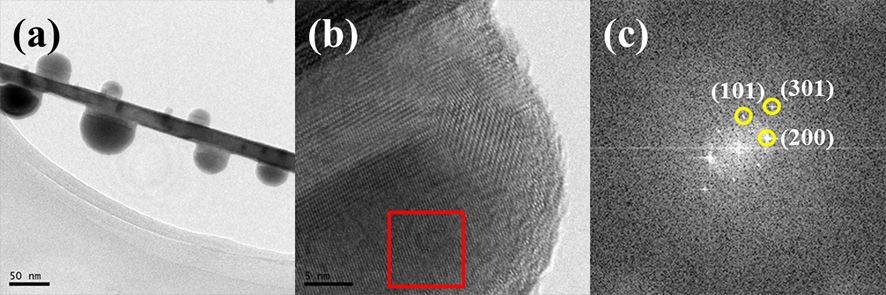


**Figure S1.** (a) Typical bright field TEM image of a Sn-decorated SnO NB, (b) high-resolution TEM image of a metal Sn nanoparticle decorated on the surface of SnO NB (c) fast Fourier transformed image corresponding to the marked square area in (b) indicating a tetragonal Sn crystal structure.

**
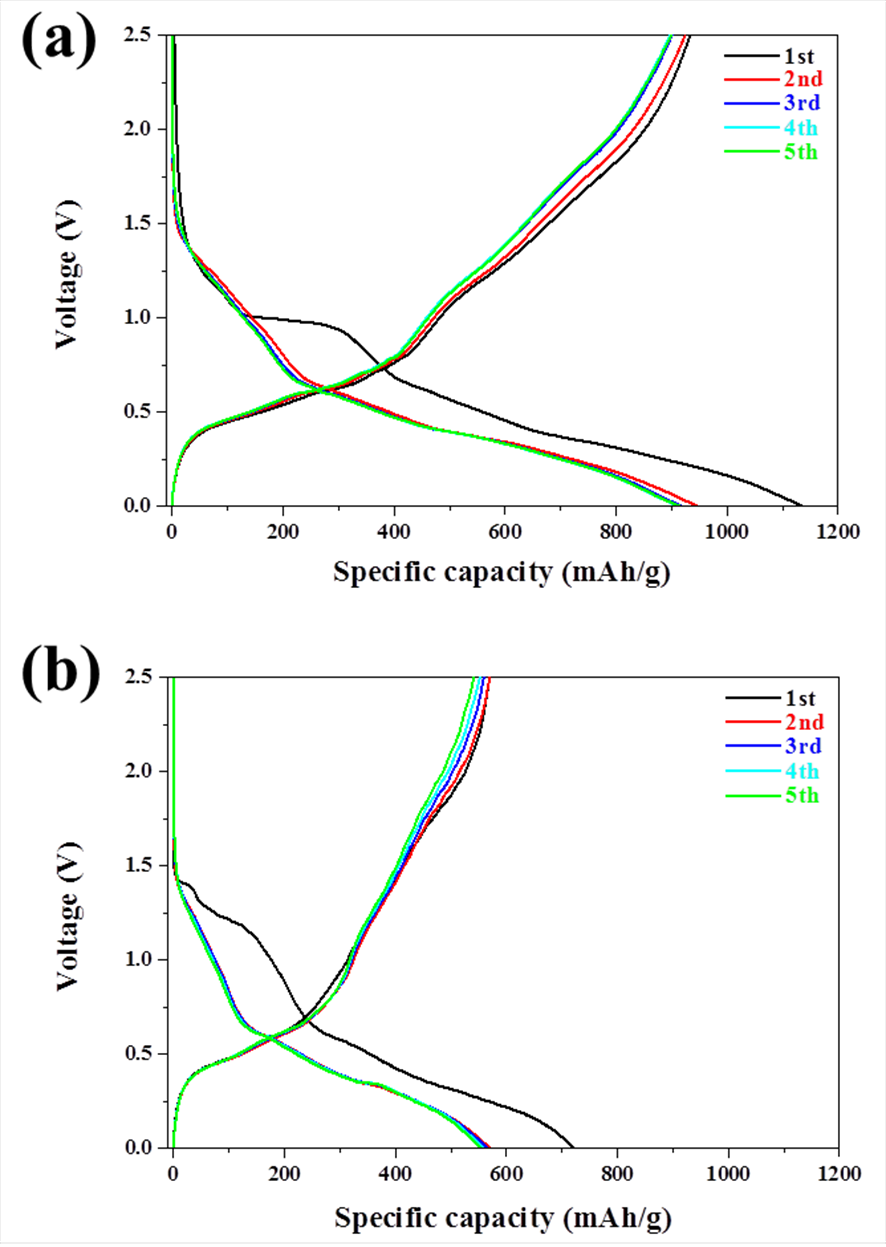
**

**Figure S2.** Charge/discharge profiles in the voltage range of 0.001 V to 2.5 V at the current density of 0.1A/g during first five cycles. (a) Sn-decorated SnO NBs and (b) SnO film.
